# On Treatment of Fissures of the Anus

**Published:** 1850-03

**Authors:** W. J. Anderson

**Affiliations:** London


*On Treatment of Fissures of the Anus.* By W. J. Anderson, Esq., London.

[The ordinary mode of treating this disease is by section of the sphincter ani. Mr. Anderson recommends this operation to be performed in a manner different from that which is usually adopted. He says:]

The operation adopted by this great surgeon, (Boyer,) and which is now generally performed, consisted in introducing the finger into the rectum, and upon it a probe-pointed bistoury, with which he cut through, "at a single stroke, the intestinal membrane, the sphincter, cellular tissue, and tegument;" thus producing a good-sized open wound. Now every surgeon must be aware that it is perfectly possible for this to be followed by a considerable amount of inflammation, and that if suppuration does occur in the loose cellular tissue around the rectum, it is an exceedingly awkward affair. This danger may be almost entirely avoided, and the operation much modified by the following plan, which I learned from the late Professor Blandin. The instrument employed for this operation consists of a sharp-pointed straight blade, with a flat sliding guard upon one side of it. This guard is rounded at the extremity, oval externally, and flat upon the surface lying on the blade.

It is compressed against the side of the knife by means of a spring, and retained in its position by a slightly projecting pin, sliding in a groove upon the blade. The whole or a portion of the guard can be retracted within the handle by a button attached to it for that purpose. The forefinger of the left hand is introduced into the rectum, and a small opening is made at the verge of the anus with the point of the knife. The guard is then protruded beyond the point, and the instrument carefully introduced through the wound into the sub-mucous cellular tissue between the mucous membrane and the sphincter ani. The cutting edge is then turned towards the muscle, the guard retracted, and the muscle cut through as the blade is withdrawn. If any doubt remains as to whether the muscle is completely divided or not, the instrument, guarded as before, may be re-introduced and a second cut made in a smaller manner exactly upon the first. But if Boyer could cut through all the tissues, in his operation, at one stroke, surely we may do it also, especially when we have not the intestinal membrane and tegument to deal with. The small external wound should then be closed, and the bowels kept in a lax state by means of the confection of senna, given in sufficient quantities to render the motions moist; but purging should be carefully avoided. It may be as well to apply some unctuous substance locally, to prevent any irritation from the feculent matter.

We have here, at any rate, an operation far less repulsive and formidable to the feelings of the patient, and certainly, in a practical point of view, attended with much less risk. The relief is as instantaneous as in the other operation; but no large open wound is left in the intestine; defectation can, therefore, be performed without the same amount of irritation; and the cure is materially expedited, inasmuch as the parts are not kept in a state of disunion longer than is necessary for its completion. Of course the same preliminary treatment, with regard to unloading the bowels, is necessary in this as in the other operation; and those excellent remarks by Sir Benjamin Brodie, concerning the impropriety of cutting directly forwards in the female, or directly backwards in either sex, are equally applicable to both methods of operating.

*Lancet, Sept.* 15, 1849, *p.* 291.

